# Supplementary material for: Plant Conservation vs. Folk Traditions: The Case of Ophrys scolopax Cav. (Orchidaceae) in Central Western Spain
Source: Biology (Basel). 2022 Oct 25;11(11):1566. doi: 10.3390/biology11111566 (PMC9687785; doi:10.3390/biology11111566)
Supplement: Supplementary file 1 [file biology-11-01566-s001.zip › biology-1974003-supplementary.pdf]

GÉNERO .... H ☐ M ☐ / EDAD .... años

NIVEL DE ESTUDIOS .... / LUGAR DE NACIMIENTO ....

1.- ¿Conoce esta planta? .... SÍ ☐ NO ☐

2.- ¿Cómo se llama? ....

3.- ¿Por qué recibe ese nombre? .... n.s./n.c.

....

4.- ¿Por qué o para qué tiene esta forma? .... n.s./n.c.

....

5.- ¿A qué grupo de plantas pertenece? .... n.s./n.c.

Fam. ORQUÍDEAS ☐ Otros: ....

6.- ¿Dónde crece esta planta en Villarino de los Aires? ....

7.- ¿En qué época del año florece? ....

8.- ¿Cómo la conoció?, ¿desde cuándo la conoce? ....

....

9.- ¿Recoge, o ha recogido, esta planta? .... SÍ ☐ NO ☐

10.- ¿Cómo la recoge, o ha recogido? ....

11.- ¿En qué cantidad la recoge, o ha recogido? ....

12.- ¿Por qué la recoge, o ha recogido? ....

....

13.- ¿Qué hace con los especímenes recogidos? ....

....

14.- ¿La recoge, o ha recogido, el Lunes de Pascua en el Teso de San Cristóbal? ....

....

15.- ¿Qué otro/s días la recoge, o ha recogido? ....

....

16.- ¿Conoce los poemas de Arturo Santos Cordero o Victoria Benito dedicados a ella? ...... SÍ ☐ NO ☐

17.- ¿Conoce la obra pictórica de Neil Allen ("el inglés") con la que ha ganado un muy prestigioso premio internacional? .... SÍ ☐ NO ☐

Figure S1. Questionnaire compiled to analyse how folk traditions of Villarino de los Aires (Salamanca, Spain) affected the bee orchid *Ophrys scolopax* in the recent past.

Table S1. Socio-demographic characteristics of the respondents (n = 242).

Variebales	Frecuency (males/females)	%
**Gender**		
Male	121	50
Female	121	50
**Age**		
< 10 years	10 (4/6)	4
10—19	22 (14/8)	9
20—29	16 (10/6)	7
30—39	20 (11/9)	8
40—49	19 (11/8)	8
50—59	34 (16/18)	14
60—69	45 (24/21)	19
70—79	41 (17/24)	17
80—89	22 (7/15)	9
90—99	13 (7/6)	5
**Educational status**		
Primary	161 (72/89)	67
Secondary (and Higher)	81 (49/32)	33
